# Large-Scale Profiling of RBP-circRNA Interactions from Public CLIP-Seq Datasets

**DOI:** 10.3390/genes11010054

**Published:** 2020-01-03

**Authors:** Minzhe Zhang, Tao Wang, Guanghua Xiao, Yang Xie

**Affiliations:** 1 Quantitative Biomedical Research Center, Department of Clinical Sciences, University of Texas Southwestern Medical Center, Dallas, TX 75390, USA; minzhe.zhang@utsouthwestern.edu (M.Z.); Tao.Wang@UTSouthwestern.edu (T.W.); guanghua.xiao@utsouthwestern.edu (G.X.); 2 Harold C. Simmons Comprehensive Cancer Center, UT Southwestern Medical Center, Dallas, TX 75390, USA; 3 Center for the Genetics of Host Defense, University of Texas Southwestern Medical Center, Dallas, TX 75390, USA; 4 Department of Bioinformatics, University of Texas Southwestern Medical Center, Dallas, TX 75390, USA

**Keywords:** Circ-RNA, CLIP-Seq, RBP

## Abstract

Circular RNAs are a special type of RNA that has recently attracted a lot of research interest in studying its formation and function. RNA binding proteins (RBPs) that bind circRNAs are important in these processes, but have been relatively less studied. CLIP-Seq technology has been invented and applied to profile RBP-RNA interactions on the genome-wide scale. While mRNAs are usually the focus of CLIP-Seq experiments, RBP-circRNA interactions could also be identified through specialized analysis of CLIP-Seq datasets. However, many technical difficulties are involved in this process, such as the usually short read length of CLIP-Seq reads. In this study, we created a pipeline called Clirc specialized for profiling circRNAs in CLIP-Seq data and analyzing the characteristics of RBP-circRNA interactions. In conclusion, to our knowledge, this is one of the first studies to investigate circRNAs and their binding partners through repurposing CLIP-Seq datasets, and we hope our work will become a valuable resource for future studies into the biogenesis and function of circRNAs.

## 1. Introduction

Circular RNAs (circRNAs) are a mysterious type of RNA that was discovered more than 30 years ago. Thought to be junk and mistakes in splicing before, now they have been demonstrated to be a class of abundant, stable and ubiquitous RNAs through mining of large-scale high-throughput RNA-Seq data and experimental validation [1,2]. Still little is known about the formation of circRNAs, while several characteristics are relatively clear: (1) they are formed when the 3’ and 5’ ends of part of the linear transcript are joined, usually with the joining points flanked by GU/AG splicing signal. (2) reverse complementary sequences in flanking introns are necessary for the formation of some circRNAs, since they can bring two discontinuous regions of RNAs close together for splicing and joining, but may not for some circRNAs [3]. (3) splicing factors regulate the formation of circRNAs [3,4]. In addition to their formation, the understanding of the functions of circRNAs is also lacking. Most circRNAs are found in cytoplasmic fractions [5] and do not have a poly A tail, though one circRNA was recently found to be translatable (4). Ashwal-Fluss et al. found that circRNAs can compete with pre-mRNA splicing [6]. Conn et al. and You et al. reported that the expression of circRNAs is regulated during Epithelial–mesenchymal transition (EMT) [7] and neuronal development [8]. Li et al. found a special class of circRNAs that can regulate transcription in the nucleus [9]. Bachmayr-Heyda et al. discovered a negative correlation of global circRNA abundance with proliferation in human samples [10]. cDR1as and Sry are the most well-known examples of circRNAs that act as miRNA sponges [11]. Nonetheless, the functions of the majority of circRNAs are still not well elucidated.

To understand the formation and functions of circRNAs, studying the RBPs that they are associated with is essential. CLIP-Seq is a technology that combines Cross-linking immunoprecipitation (CLIP) [12] and next-generation sequencing (NGS), and it has already greatly improved our understanding of RBP-RNA interactions at the genome scale [13]. HITS-CLIP [14], PAR-CLIP [15] and iCLIP [16] are the three main variants of CLIP-Seq that have been extensively used to identify RBP binding targets, and this has led to deep insights into cellular mechanisms and disease etiology. eCLIP [17], irCLIP [18], and sCLIP [19] are recent advancements in CLIP-Seq technology, and were intended to overcome the low complexity and specificity problem of early generation protocols. Although they differ with respect to their cross-linking strategies and library preparation procedures, all these techniques purify and gather RNA sequencing reads, overlapping the binding position of RBPs, which should theoretically also include those from circRNAs. In this regard, if sequencing reads are observed to be mapped across the splicing joining sites of circRNAs, it would provide direct evidence of RBPs’ binding to circRNAs. A few studies have explored the possibility of using CLIP-Seq datasets to identify circRNA-RBP interactions. For example, Li et al. conducted Pol II CLIP-Seq and revealed a subclass of nucleus-located circRNAs that are associated with Pol II [9]. Conn et al. used PAR-CLIP to show that Quaking regulates the formation of circRNAs via binding sites in introns [7]. However, to our knowledge, a systematic analysis of public CLIP-Seq datasets for this purpose has not yet been presented.

To achieve this task, CLIP-Seq reads need to be filtered to select those that support the existence of circRNAs. Guo et al. developed a pipeline and applied it to ENCODE data [5]. CIRI is another software to detect circRNAs from transcriptome data [20,21]. circBase is a database that merges datasets of circRNAs from different organisms [22]. The rationales behind these works are similar: an RNA-Seq read whose 3′ end and 5′ end are mapped to the upstream and downstream of a transcript, respectively, in a reverse configuration is evidence of circRNAs, and non-poly A selected paired-end RNA-Seq data is the most suitable for this task. This strategy should also guide the discovery of circRNAs in CLIP-Seq data. However, directly applying the previous pipelines developed on RNA-Seq data to CLIP-Seq data would be difficult due to three major challenges: (1) CLIP-Seq technology involves enzymatic digestion, leading to generally very short read length; (2) CLIP-Seq data are almost exclusively single-end; (3) CLIP-Seq data usually have limited library complexity, yielding a high PCR duplicate rate. CIRI, for example, is known to have a high false discovery rate for single-ended data, which refuses to process data whose alignment length is smaller than 40 nt and cannot distinguish between PCR duplicates.

In this study, we created a pipeline called Clirc, specialized in profiling circRNAs in CLIP-Seq data, and we applied Clirc to collecting public CLIP-Seq datasets to detect circRNAs bound by RBPs, and analyzing the characteristics of RBP-circRNAs interactions. We focused on HITS-CLIP, PAR-CLIP and iCLIP data, as they have the most abundant public sources and share similar data characteristics compared to newer versions of CLIP-Seq data.

## 2. Materials and Methods

### 2.1. Downloading CLIP-Seq Datasets

We downloaded 167 CLIP-Seq datasets from GEO and other data repositories whose RBPs are wild-type and whose experimental conditions are controlled. These RBPs are from Human, Mouse and Drosophila Melanogaster (metadata of all the datasets information is provided in Supplementary File S1). Low-quality CLIP-Seq datasets were discarded after FastQC quality examination. The adaptor sequences were found by reading experimental protocols, FastQC detection or manual comparison. After trimming adaptors, CLIP-Seq reads that were too short were discarded from the analysis. To tackle the high PCR duplicate rate problem caused by limited library complexity, the remaining reads with exactly the same nucleotide sequence were collapsed to unique tags.

### 2.2. Linearization of circRNA Library

We collected a series of published literature that identified circRNAs by mining RNA-Seq data in Human, Mouse, and Drosophila (Supplementary Table S1) [23,24,25]. We also ran CIRI2 on Encode non-poly A selected paired-end RNA-Seq data to discover more circRNAs. We did this for 15 Drosophila RNA-Seq examples and 16 Mouse samples (Supplementary Table S1). We pooled the circRNAs for each species together. The low complexity and repetitive regions were identified and counted in each circRNA, if the length of such regions exceeded a certain proportion of the whole circRNA length, it would be discarded. Then in each circRNA, the RNA sequence that started from 50 bp upstream of the junction site and ended at 50 bp downstream of the junction site was extracted and added as an artificial chromosome to the reference genome (we recommend the version in which repetitive/low-complexity regions are masked). Then the index building functionality of the gsnap software [26,27] was used to build a combined index file for the following alignment.

### 2.3. Competitive Alignment of CLIP-Seq Reads

For each study, CLIP-Seq reads were mapped simultaneously to the linearized circRNA library and the normal reference genome by alignment to the combined index file. One read can be aligned to 0, 1, or more places in either the normal reference genome or circRNAs. A read was said to be truly aligned to a circRNA only if its alignment coordinates extended more than 5 bp away from the junction site in both directions, and the overall mismatch rate of overhang region in either direction was smaller than 0.15. Of all CLIP-Seq reads, the only ones that can be uniquely aligned to circRNAs would be assigned to circRNAs. Reads aligned to both circRNAs and linear transcripts or aligned to multiple circRNAs are discarded.

### 2.4. Filtering for CLIP-Seq Reads Supporting RBP-circRNA Interactions

After screening for potential circRNA-supporting reads from one or more CLIP-Seq samples in the same condition for the same protein, all such reads were pooled and grouped by the circRNAs they are mapped to. Then, a series of filters was applied to keep high-confidence circRNAs that were bound by each RBP: (1) only circRNAs that had at least 2 CLIP-Seq reads mapped across their junctions were kept; (2) only when at least 20 nucleotides on a circRNA were covered by at least one junction-spanning CLIP-Seq read would this circRNA be kept; (3) for all the CLIP-Seq reads mapped across a circRNA junction region, the number of unique mapping start and end positions must both be no less than 1/3 of the reads number. Only when a circRNA met all three criteria would it be retained and declared to be a candidate circRNA bound by the corresponding RBP. Clirc does not internally consider background signals, as usual CLIP-Seq data do not generate control samples, and general RNA-Seq samples are not too valuable for background control since most of them are poly A selected. However, if a non-poly A selected matched RNA-Seq data is available, users can always apply Clirc to CLIP-Seq and control samples respectively and remove the recurrent circRNAs in both conditions. This should be easy as Clirc keeps all the intermediate outputs.

### 2.5. Software Implementation

Clirc is a user-friendly software that implements the above-mentioned pipeline in Perl language. The alignment and filtering rules are based on the default parameters for the Clirc software (Quantitative Biomedical Research Center, University of Texas Southwestern Medical Center, Dallas, TX, USA), but all of them are tunable to serve users’ needs. The Clirc software depends on the gsnap aligner for circRNA analysis. The software and user manual can be accessed at https://github.com/Minzhe/Clirc.

### 2.6. Motif Search

A HOMER motif search [28] was carried out on the circRNA sequences that were covered by at least one supporting CLIP-Seq reads. To create a stringent control, we found pairs of neighboring exons in each species and concatenated the last 15 bp of the upstream exon with the first 15 bp of the downstream exon to create a library of background RNA sequences representing junction reads across splice sites.

### 2.7. “Strand Bias” of RBP Binding to circRNAs

CircRNAs are usually embodied within regular linear genes that are called the parental genes of the respective circRNAs. These genes can be transcribed in either the sense or antisense directions, which may later produce circRNAs. RBPs can interact with circRNAs derived from different strands, and the relative abundance (proportion of reads from sense strands in all reads), which we term as “strand bias” may vary among different RBPs, though bias towards sense strand is expected. In this study, we calculated the “strand bias” of detected circRNAs and 3000 sampled linear transcripts bound by an RBP, and investigated whether they were of difference.

### 2.8. Hierarchical Clustering of RBPs

Hierarchical clustering of RBPs was conducted based on their circRNA binding profiles. Distance matrix was calculated by looping every possible pair of RBPs and get a hypergeometric test *p*-value of the commonality of the circRNAs species they bound to. The top 50 most frequently occurring circRNAs (related to Supplementary Figure S1) were discarded in the analysis.

### 2.9. Gene Ontology Enrichment Analysis

Gene ontology (GO) terms were downloaded from GSEA [29] website using all curated gene sets category. Go terms with less than 10 genes were discarded. The top 50 most frequently occurring circRNAs (related to Supplementary Figure S1) were also discarded in the analysis. We calculated the hypergeometric test *p*-value (log transformed) for whether the set of parental genes for circRNAs bound by each RBP was enriched in any GO ontology term. As a background control, we randomly sampled a set of genes with the same size as the set of parental genes for each RBP and calculated a background *p*-value for each GO term. We subtracted the averaged background *p*-values from 10 randomizations from the true test *p*-value for each RBP and each GO term. We controlled the significance level at 0.05 and adjusted for multiple comparison correction with the Bonferroni method.

## 3. Results

### 3.1. The Implementation of the Clirc Software

**Figure 1:**
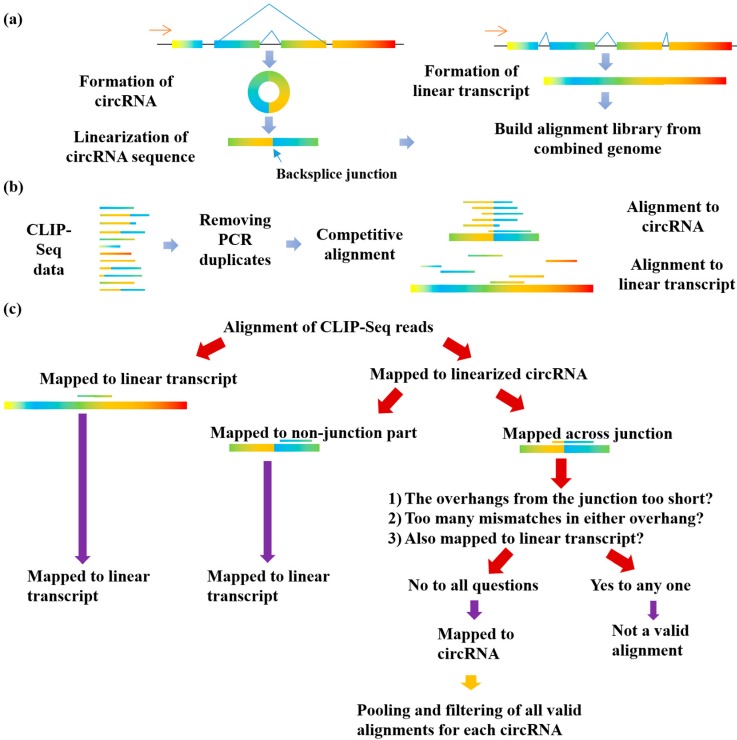
Cartoon of the Clirc pipeline to identify circRNAs bound by RBP from CLIP-Seq data. (**a**) Build combined library from linearized circRNA sequences and linear transcripts, (**b**) Competitive alignment of CLIP-Seq reads to combined library, (**c**) Identification of circRNAs through Clirc pipeline.

As mentioned above, CLIP-Seq reads are already very short and single-end. Common analysis methods for identifying circRNAs will truncate these short CLIP-Seq reads into even shorter segments for alignment, which could lead to ambiguous alignment to multiple places in the genome or no confident alignment at all. To avoid this problem, we tried to create a pseudo reference alignment library in which all circRNAs are represented as continuous sequences so that CLIP-Seq reads can be aligned as a whole. To achieve this, we collected the locations of previously published circRNAs on the genome-wide scale in the human, mouse and Drosophila species [6,22,30,31]. We also ran CIRI2 on the representative mouse and Drosophila Encode non-poly A selected RNA-Seq datasets. Each of these sets contains around 2000 to 90,000 circRNAs. The circRNA sets for each species are pooled and redundant circRNAs are removed. Then we compiled a pseudo reference genome by linearizing the circRNA sequence across the junction sites and combined this reference library with the normal genome. Since CLIP-Seq reads are highly redundant due to the limited library complexity, we collapsed CLIP-Seq reads with the same nucleotide sequences into unique tags before alignment, which is a common practice in CLIP-Seq data analysis [13]. We chose the gsnap aligner to align CLIP-Seq reads because of its high alignment quality with RNA-Seq data. Then, CLIP-Seq reads were aligned competitively to this combined library, and filtered to identify RBP-bound circRNAs. More details can be found in the materials and method section. Figure 1 shows the workflow of the Clirc software. Since CLIP-Seq is just a variation of RNA-Seq data, this software can be generalized to RNA-Seq data.

### 3.2. Profiling circRNAs Bound by RBPs Using Clirc

**Figure 2:**
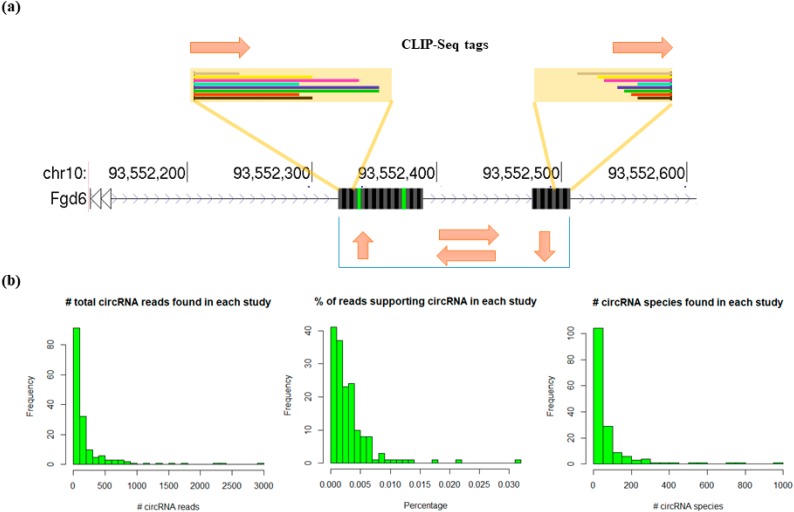
Overview of circRNAs bound by RBPs. (**a**) Exemplary circRNA bound by mouse RBP SRSF1. (**b**) Summary statistics of circRNAs bound by RBPs in all analyzed RBPs.

We downloaded 167 CLIP-Seq datasets, and all datasets we downloaded were wild-type RBPs in control conditions. These datasets included 91 human RBPs, 26 mouse RBPs, and 10 drosophilas RBPs. The human RBP CLIP-Seq datasets also included four IgG controls. We preprocessed the fastq files and applied the Clirc software to identify RBP bound circRNAs on these datasets. Figure 2a shows an exemplary circRNA found in an SRSF1 CLIP-Seq dataset in mouse embryonic fibroblasts, which spans two exons of the Fgd6 gene. To test the validity of the proposed pipeline, we applied the Clirc software to a mouse and a human cancer cell line DNA-Seq data as negative controls, and two RNA-Seq samples matched with two CLIP-Seq data in our dataset as background controls (Supplementary Table S2). The circRNA supporting reads discovery rate in DNA-Seq samples are extremely low (in 50 bp long DNA reads) or undetectable (in 100 bp long DNA reads), which suggested a good stringency of our pipeline. The mapped circRNAs reads ratios in matched RNA-Seq samples were also considerably lower than CLIP-Seq. This was probably due to the poly A selection of those samples. As a positive control, we investigated whether Clirc was able to re-identify the top 15 experimentally validated circRNAs bound by PolII in a previous report [8]. In Supplementary Table S3, we showed the number of CLIP-Seq reads identified by our algorithm that supported each circRNA. 14 of these 15 circRNAs were supported by at least two CLIP-Seq reads, showing Clirc is accurate and sensitive in identifying RBP-bound circRNAs. Figure 2b shows the summary statistics of the circRNAs found in all CLIP-Seq datasets. The Clirc pipeline found circRNA reads in most CLIP-Seq datasets, with a few studies having up to 3000 circRNA-supporting reads. Even the CLIP-Seq study with the most abundant circRNAs has less than 0.1% of all CLIP-Seq reads mapped across circRNA. However, this percentage is similar to the percentage of sequencing reads supporting circRNAs found in the Encode RNA-Seq data we used to search for circRNAs. In addition, the number of unique circRNA species found in each CLIP-Seq dataset is mostly between 0 and 200, with a few RBPs binding up to 1000 unique circRNAs. Overall, many CLIP-Seq studies yield significant amounts of circRNA-supportive reads that we could use for more detailed downstream analysis. We provided a complete list of all the circRNAs found in human, mouse and drosophila in Supplementary File S2–S4.

### 3.3. Binding Properties of RBPs on circRNAs

**Figure 3:**
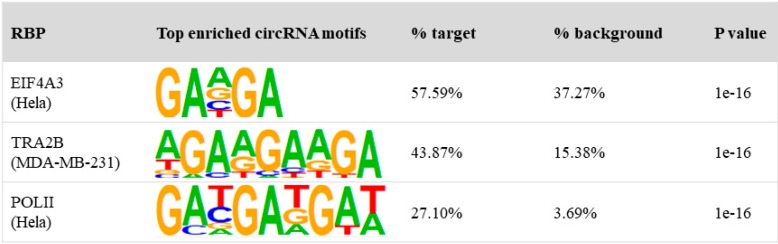
HOMER motif search results for EIF4A3, Tra2b, and Pol II. The left panel shows the protein and cell line/tissue information, the middle panel shows top circRNA motif and the right panel shows the *p*-values and percentages of sequences containing each motif. RBP, RNA binding proteins.

We investigated whether RBPs bind to circRNAs through recognition of sequence motifs. Of all CLIP-Seq studies investigated, we found a motif that seems to be enriched over the background. It is a GA-rich motif that occurred in circRNAs bound by Eif4a3, Tra2b, and Pol II (Figure 3), but which was not found in the entire circRNA library. The recurrent significant motif increased our confidence that there could be important biological functions of some motifs that mediate the interaction between RBPs and circRNAs. The GA-rich motif is very similar to the GAAGAA-like exonic splicing enhancer that is known to direct and enhance accurate splicing [32,33]. Consistent with the known function of this motif, Eif4a3 and Tra2b are previously known to be involved in alternative splicing [34,35]. Splicing is also known to be coupled to transcription by RNA polymerase II through the C-terminal domain of the RNAPII largest subunit. This may also suggest that the function of Pol II-associated circRNAs to regulate transcription could be related to alternative splicing of the linear RNA precursor. This result suggests that at least some RBPs recognize target circRNAs through sequence motifs, and that motif analysis could hint at potential functions of RBP-circRNA interactions.

**Figure 4:**
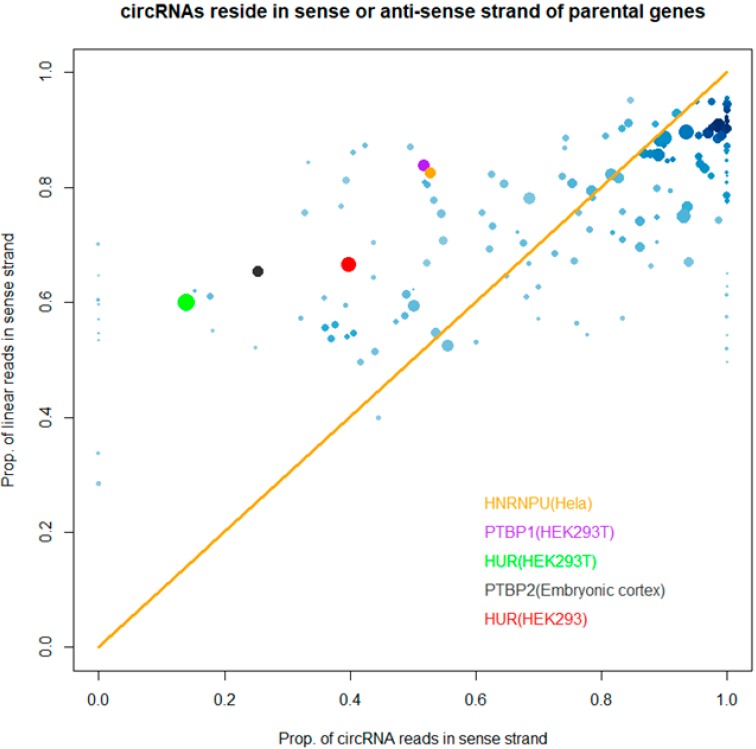
Proportion of circRNA reads in the same strand of the parental genes (x) vs. proportion of CLIP-Seq reads mapped to linear transcripts in sense strand (y). Dot size represents number of circRNA sequences in a study. The CLIP-Seq reads whose circRNA sequences are more than 500 and whose absolute (x-y) difference is more than 20% are labeled at the bottom right corner.

We also investigate d whether the “strand bias” of RBP-bound circRNAs deviates from it of linear transcripts (Figure 4). We plotted the proportions of CLIP-Seq reads mapped to linear transcripts and circRNAs in the same or opposite direction of the linear genes for different RBPs. Overall, the “strand bias” of bound circRNAs was consistent with it of linear transcripts for most studies, and bias towards sense strand was dominant. Interestingly, we observed that several RBPs showed different strand preference between linear transcripts and circRNAs. PTBP1 and PTBP2 from two different studies showed a much larger proportion of circRNA reads in the anti-sense direction of parental genes than it of linear transcripts. HUR protein from two different studies also consistently showed that it tended to bind circRNA in the anti-sense direction of parental genes. This suggested that “strand bias” of RBP-bound circRNA was a reproducible phenomenon. However, it must be pointed out that this was an observation at the global level, not at the individual gene level. At the individual gene level, transcription was still in either the sense or antisense strand, not mixed. For example, the source gene that produced the most abundant circRNAs HUR bound to was XIST. It generated 33 circRNAs, all from the sense strand. Another gene TRAM1 generated 25 circRNAs, all from the antisense strand. However, at the global level, the circRNAs of certain RBPs display bias toward the occupation of sense or anti-sense strands. Further investigation is needed to validate whether the “strand bias” is of functional importance.

### 3.4. Functional Implications of circRNA-RBP Interactions

To investigate the coordination of RBPs on circRNAs, we pooled circRNAs that appeared in all RBPs’ binding relationships, and calculated for each circRNA the percentage of all RBPs that are found to bind to this circRNA. Data for the same RBP in different cell lines or tissue types were combined in this analysis. We showed the most common 100 circRNAs in Supplementary Figure S1, together with whether these circRNAs appeared in the 4 IgG control experiments. It appears that the top subset of circRNAs (e.g., top 20) found to bind RBPs are also more likely to be found in the IgG experiments, suggesting that these circRNAs may be non-specific binding targets. Echoing this observation, it has been reported before that some very abundantly expressed RNAs are commonly represented in CLIP-Seq datasets [30], but they could just be artifacts that are not truly bound by the RBPs. However, there are numerous circRNAs that are not in the top set but are also bound by multiple RBPs, and these circRNAs could truly serve as platforms for binding and coordination of multiple RBPs.

**Figure 5:**
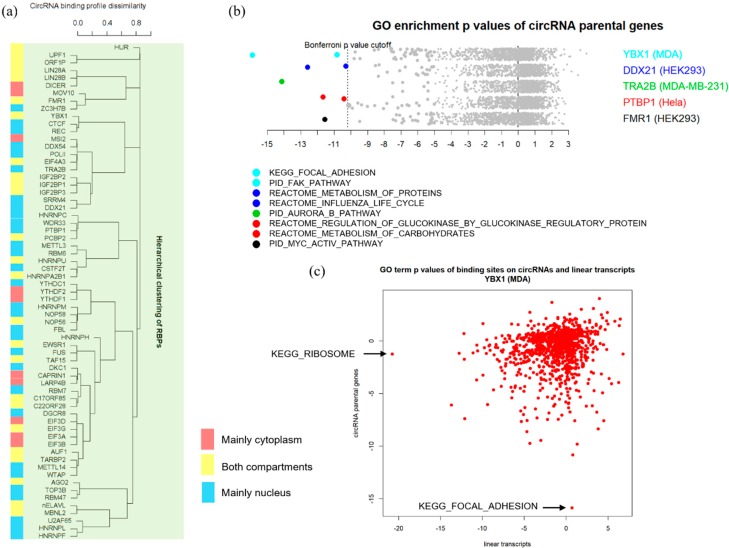
Functional implications of RBP-circRNA interactions. (**a**) Hierarchical clustering plot of RPBs based on the similarity of their circRNA binding profile. This analysis is only conducted for the human RBPs. Red marks RBPs that are mainly cytoplasmic, yellow marks RBPs that abundantly exist in both cytoplasm and nucleus, and blue marks RBPs that are mainly nuclear. (**b**) Enriched gene ontology (GO) terms for each RBP. Log-transformed *p*-value is calculated as *p*-value from a hypergeometric test of each set of parental genes of circRNAs bound by an RBP vs. genes in each gene ontology term, subtracted by averaged random *p*-value from hypergeometric tests of 10 randomizations. The significance level is controlled at 0.05, adjusted by the Bonferroni method. Significantly enriched GO terms are labeled according to the colors of the RBPs. (**c**) Log-transformed GO terms enrichment *p*-values of binding sites in circRNA parental genes vs. in linear transcripts for the YBX1 protein. The adjustment method is the same.

To further explore the possibility of circRNAs serving to coordinate RBPs, we tried to find whether RBPs that bind similar circRNAs share similar properties. We conducted a hierarchical clustering of RBPs based on the similarity of their circRNA binding profile (Figure 5a). This analysis was done only in human protein. In the plot, RBPs that were clustered more closely together tend to bind a similar set of circRNAs and vice versa. Then we labeled the RBPs on this plot by their cellular localization annotation according to UniProt [36]. We categorized RBPs into 3 types, RBPs that are mainly in the cytoplasm, RBPs that exist abundantly in both cytoplasm and nucleus, and RBPs that are mainly in the nucleus. Interestingly, we observed a trend that RBPs that belong to the same category clustered more closely together than RBPs in different categories. This trend will be even more obvious if we combine the first and second category to compare the difference between nucleus-only RBPs and other RBPs. This result seems to partially support previous conjectures that circRNAs may act as a scaffold to bind and sequester multiple RBPs [37].

Finally, we investigated whether the parental genes for circRNAs bound by each RBP show enrichment in certain biological functional categories. We only did this for human RBP binding circRNAs deriving from more than 100 distinct parental genes. Among all 1329 GO terms, only for some terms did a few RBPs result in appreciably significant *p*-values (Figure 5b, Supplementary Table S4). We also compared the logarithm GO enrichment *p*-value of the parental genes of circRNAs with linear transcripts, and no apparent correlation was found. Interestingly, the most significant ontology term observed was “KEGG_FOCAL_ADHESION”, found in the YBX1 protein in the MDA cell line. However, it was not enriched at all in the linear transcript. On the contrary, the most enriched GO term for the linear transcript was “KEGG_RIBOSOME”, but not enriched in circRNAs (Figure 5c). YBX1 is known to participate in pre-mRNA alternative splicing. Recently, several studies also reported that YBX1 was involved in cell adhesion and mediated resistance to focal adhesion kinase (FAK) inhibitor in cancer [38,39]. For another protein, DDX21, the most significant gene ontology term was “REACTOME_INFLUENZA_LIFE_CYCLE” (Supplementary Figure S2). According to the literature, one recently known function for DDX21 is inhibition of influenza A virus replication [40]. These results seem to suggest that for at least some RBPs, their bound circRNAs might mediate their cellular functions, but more research needs to be carried out to validate this hypothesis and the implications of the significant ontology terms of other RBPs’ bound circRNAs.

## 4. Discussion

This study is the first to our knowledge to identify RBP binding sites on mature circRNAs systematically using all public CLIP-Seq datasets. The bioinformatics approach employed here will give an unambiguous answer to whether there is a direct interaction between an RBP and a circRNA in contrast to the linear form. This will shed light on both the formation and function of circRNAs, both of which are understudied for the moment. We carried out a series of analysis in this study to characterize the basic properties of circRNA-RBP interactions, including motif patterns and gene ontology enrichment. Our pipeline was wrapped into the Clirc software so that other researchers could use it to conveniently investigate RBP-bound circRNAs in their future CLIP-Seq studies.

Due to the certain level of error rate inherent in high-throughput sequencing, some chimera reads that do not represent real circRNAs could be identified as false positives by the Clirc software. Recognizing this risk, our analysis and the Clirc software was intentionally designed to be conservative. First of all, the set of circRNAs that were linearized to be combined with the reference genome were published in previous studies or found by CIRI2 on Encode RNA-Seq data. The size of the circRNA set from each source ranges from 2000 to 90,000 (Supplementary Table S1), some of which are probably false positives. Clirc essentially scans through each CLIP-Seq study to find CLIP-Seq reads that can be aligned to these previously defined circRNAs. Therefore, the resulting RBP-bound circRNAs come from the intersection of the RNA-Seq data and the CLIP-Seq data, which is supposed to significantly decrease false positive rate. On the other hand, Clirc involves a series of stringent filters to narrow down the list of CLIP-Seq reads supportive of circRNAs. In Supplementary Table S3, although these 15 circRNAs are all known to be real and bound by Pol II, Clirc only identifies 14 of them, with the remaining one missed due to abundant low complexity/repetitive sequence regions. On balance, Clirc was designed to be rigorous in calling RBP-bound circRNAs, even at a slight sacrifice of a higher false negative rate.

Integrative analysis of CLIP-Seq data with other types of high-throughput data types can be a very interesting research direction but has so far not been intensively explored yet. One recent study [41] identified 22,735 RBP-lncRNA regulatory relationships from more than 100 public genome-wide CLIP datasets. This study serves as an example of how integrative analysis could lead to meaningful discoveries. In this study, we investigated the possibility of integrative analysis of CLIP-Seq datasets with circRNA data to characterize the function of RBP-circRNAs interactions. In the future, it could also be interesting to integrate CLIP-Seq data-derived circRNA-RBP interactions with The Cancer Genome Atlas (TCGA) data. The Non-Coding RNA Explorer [42] has been trying to document circRNAs identified in different types of tumors using TCGA RNA-Seq data. The importance of circRNAs and their interacting RBPs for prognostic survival prediction and influence on treatment efficacy may be investigated by leveraging this resource.

There are a few limitations and pitfalls of this study: (1) Clirc detects RBP-circRNA binding events only in the junction site, and is not capable to detect binding events in non-junction regions as they are not distinguishable from those occur in the linear transcript, though also important. For example, during the formation of circRNAs, the upstream and downstream introns surrounding the circRNAs will form a “stem”, in which RBPs, especially splicing factors, may play a role. Such events cannot be investigated in this study. (2) Clirc identifies RBP bound circRNAs by competitively aligning reads to both normal reference genome and a pseudo reference genome generated with known circRNAs. It is designed to detect only circRNAs that are presented in the library but not novel ones. Therefore, the sensitivity of Clirc demands the comprehensiveness of circRNA library. To achieve this, we collected thousands of existing circRNAs from previously published studies and databases, and supplemented it by running CIRI2 exhaustively on all suitable paired-end non-poly A selected Encode RNA-Seq data. (3) Usually CLIP-Seq data does not generate control samples, and occurrence of junction spanning reads is also low, so the peak finding procedure is not applicable. Clirc may still have a small chance to falsely align non-supportive reads to the junction region or identify transient RBP-circRNA interaction by low abundance of supportive reads, although stringent rules have been applied to filter out those false positives.

## 5. Conclusions

Overall, we created the Clirc pipeline to identify RBP-bound circRNAs from CLIP-Seq datasets. Through competitive alignment and additional filtering, Clirc searches for back-splice junction spanning reads an evidence for circRNAs. We used this pipeline to identify RBP-bound circRNAs in CLIP-Seq datasets in the public domain and we also characterized the RBP-circRNA interactions from a genome-wide perspective. We hope this novel approach will contribute to the circRNA research community and broaden our knowledge of transcription and its regulation in the long run.

## Supplementary Materials

The following are available online at https://www.mdpi.com/2073-4425/11/1/54/s1, Figure S1: Binding profiles of the top 100 circRNAs bound by most RBPs, Figure S2: GO terms p values of binding sites on circRNAs and linear transcripts of DDX21 in HEK293 cell, Figure S3: Hierarchical clustering plot of RPBs based on the commonality of their circRNA binding profile, Table S1: CIRI2 analysis and previous publications that provided large scale existence and locations of circRNAs, Table S2: Number of circRNA supporting reads identified by Clirc in CLIP-Seq, matched RNA-Seq, and DNA-Seq samples, Table S3: Number of CLIP-Seq reads identified by Clirc to support each of the 15 most enriched PolII-associated circRNA that have been experimentally validated before, Table S4: The significant GO term for the parental genes of each RBP’s bound circRNAs, File S1: metadata of all the datasets information, File S2, S3, and S4: a complete list of all the circRNAs found in human, mouse and drosophilas, respectively.
