# Supplementary material for: Large-Scale Profiling of RBP-circRNA Interactions from Public CLIP-Seq Datasets
Source: Genes (Basel). 2020 Jan 3;11(1):54. doi: 10.3390/genes11010054 (PMC7016857; doi:10.3390/genes11010054)
Supplement: Supplementary file 1 [file genes-11-00054-s001.zip › genes-657053-suppl/Supplementary figures and tables.docx]

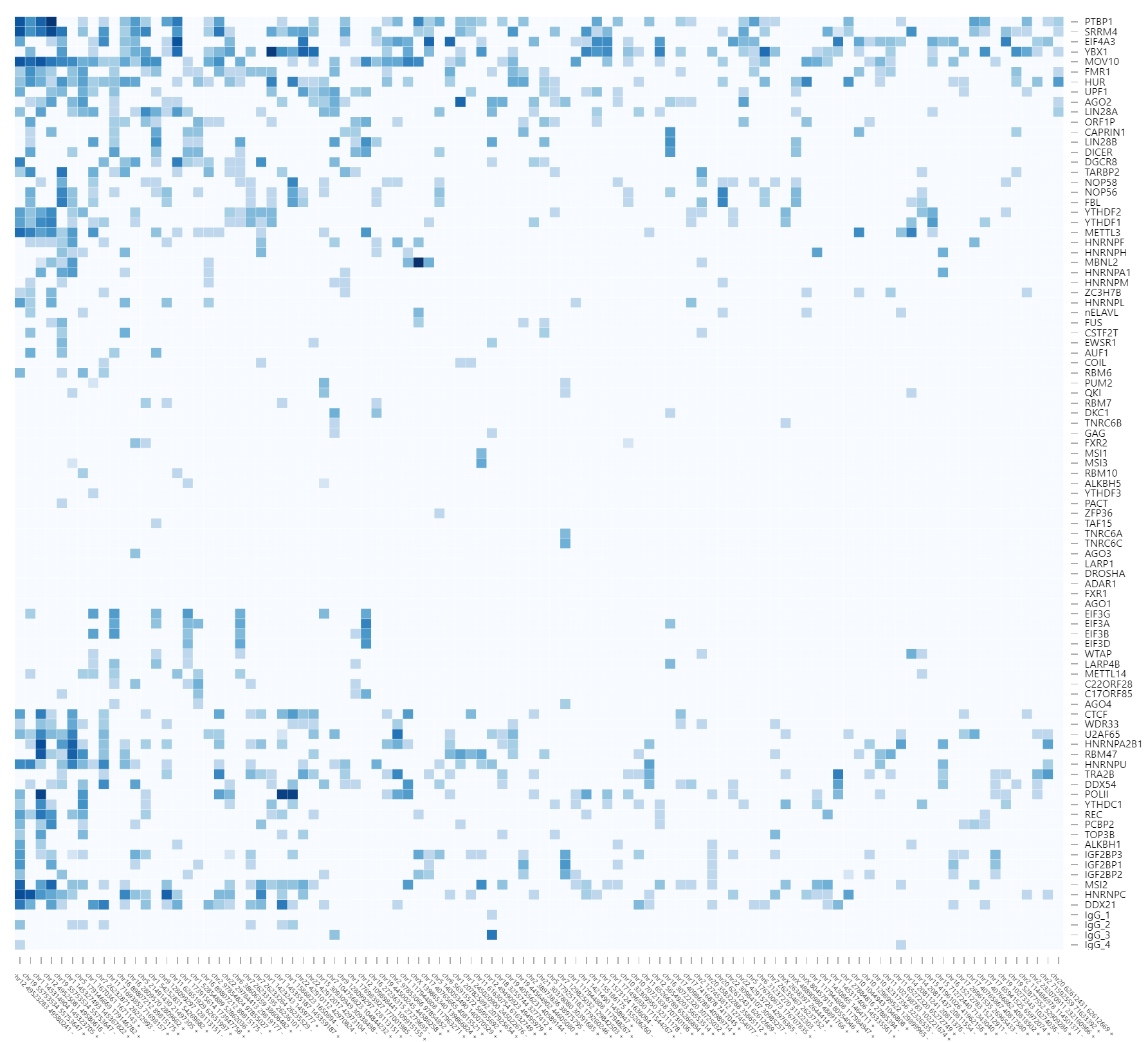


**Sup. Fig. 1** Some circRNAs are discovered in the binding target sets of multiple RBPs. Each row is one RBP (multiple CLIP-Seq datasets on the same protein were merged) or one of the 4 IgG controls. Each column is one of the top 100 circRNAs (top 1 to 100 from left to right) that appeared the most number of times in all RBPs. Color represents the value of log(# of supporting reads + 1). Darker color means more supporting reads found.

**
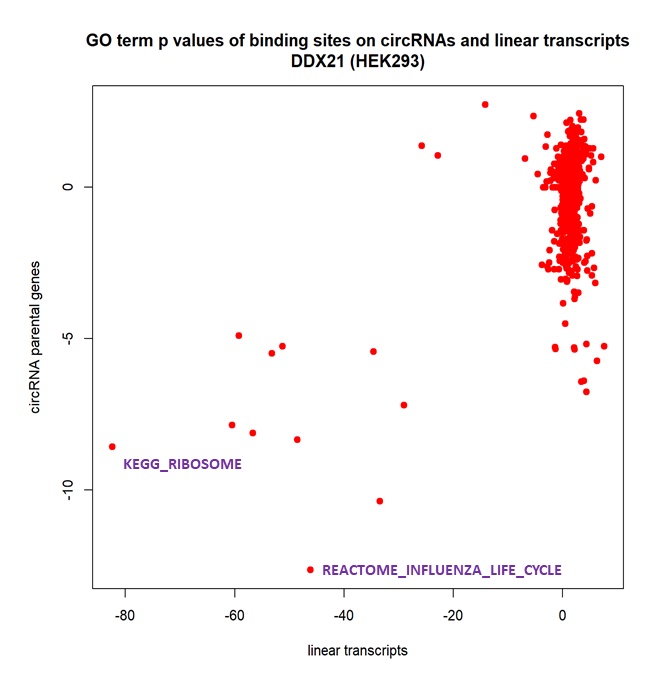
**

**Sup. Fig. 2** GO terms enrichment P values of binding sites in circRNA parental genes vs. in linear transcripts for the DDX21 protein. The adjustment method is the same as that mentioned in **Fig. 4b**.

**
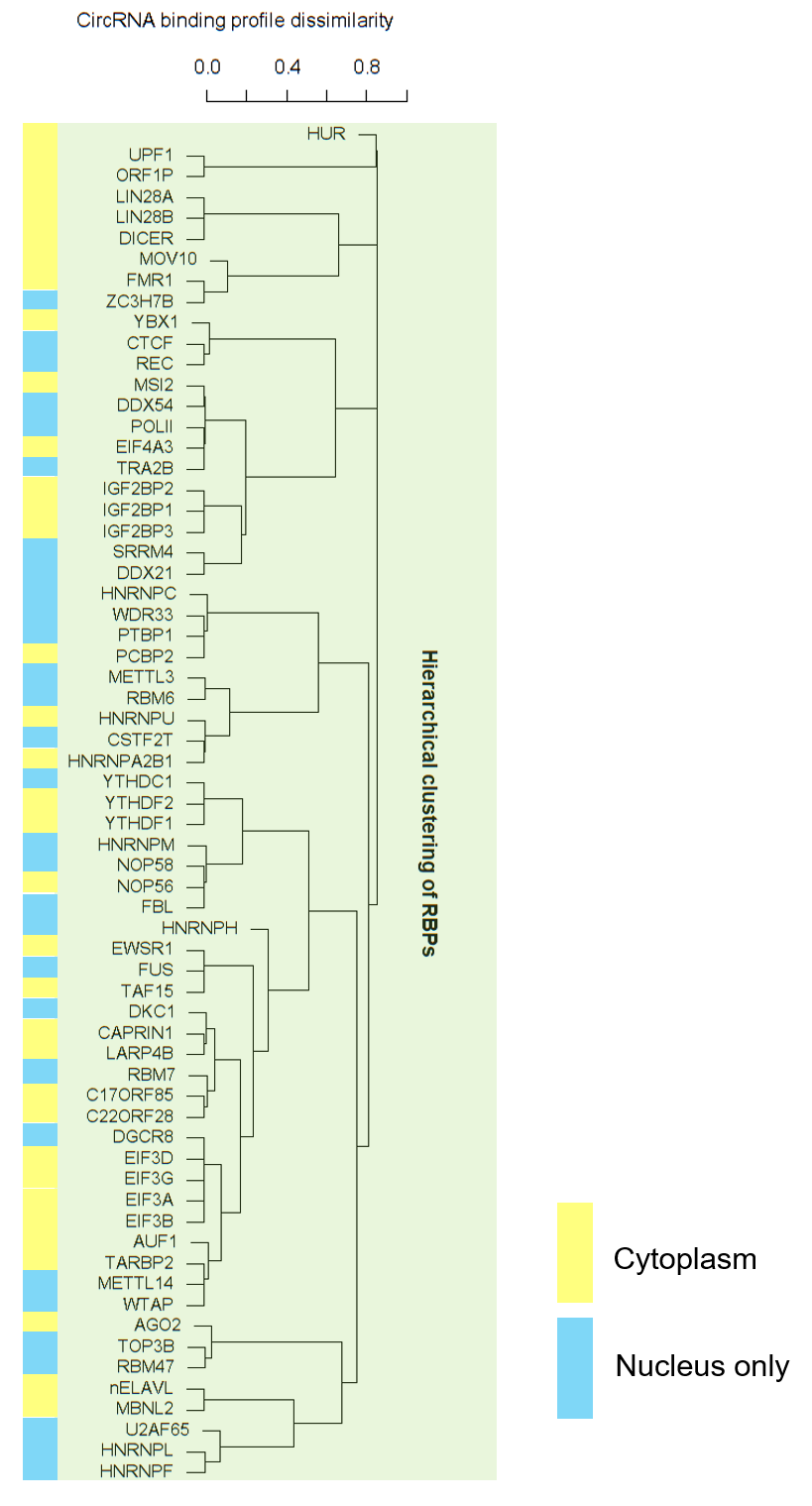
**

**Sup. Fig. 3** Hierarchical clustering plot of RPBs based on the similarity of their circRNA binding profile. This analysis is only conducted for the human RBPs. Yellow marks RBPs that are mainly cytoplasmic or abundantly exist in both cytoplasm and nucleus, and blue marks RBPs that are mainly nuclear.

**Sup. Table 1** CIRI2 analysis and previous publications that provided large scale existence and locations of circRNAs

| Source | Species | # circRNAs |
| --- | --- | --- |
| Ashwal-Fluss et al. (6) | Drosophila | 4,053 |
| Westholm et al. (28) | Drosophila | 38,115 |
| CIRI2 analysis on Encode data | Drosophila | 1,466 |
| Rybak-Wolf et al. (29) | Human | 65,731 |
| Glazar et al. (22) | Human | 92,375 |
| Gao et al. (20) | Human | 11,390 |
| Rybak-Wolf et al. (29) | Mouse | 15,849 |
| Glazar et al. (22) | Mouse | 1,903 |
| CIRI2 analysis on Encode data | Mouse | 6,837 |

**Sup. Table 2** Number of circRNA supporting reads identified by Clirc in CLIP-Seq, matched RNA-Seq, and DNA-Seq samples.

| Species | Dataset | RBP | Library | Number of mapped reads | | |
| --- | --- | --- | --- | --- | --- | --- |
|  |  |  |  | **Total** | **CircRNA** | **CircRNA ratio** |
| Mouse | GSE60487 | MBNL1 | CLIP-Seq | 4032433 | 24 | 0.00060% |
|  |  | MBNL2 | CLIP-Seq | 4092634 | 64 | 0.00156% |
|  |  | MBNL3 | CLIP-Seq | 509225 | 2 | 0.00039% |
|  |  | N/A | RNA-Seq | 26378217 | 13 | 0.00005% |
| Human | GSE46705 | METTL3 | CLIP-Seq | 2487710 | 73 | 0.00293% |
|  |  | METTL14 | CLIP-Seq | 2069050 | 54 | 0.00261% |
|  |  | WTAP | CLIP-Seq | 1829461 | 40 | 0.00219% |
|  |  | N/A | RNA-Seq | 27152698 | 4 | 0.00002% |
| Mouse | B-16 WES | N/A | DNA-Seq | 41100044 | 12 | 0.00003% |
| Human | H2009 WES | N/A | DNA-Seq | 73737463 | 19 | 0.00003% |

**Sup. Table 3** Number of CLIP-Seq reads identified by Clirc to support each of the 15 most enriched PolII-associated circRNA that have been experimentally validated before.

| circRNA name | Chromosome | Start | End | Strand | # supporting reads |
| --- | --- | --- | --- | --- | --- |
| circEIF3J | chr15 | 44843074 | 44843720 | + | 3 |
| circPAIP2 | chr5 | 138699448 | 138700432 | + | 2 |
| circRSRC1 | chr3 | 157839892 | 157841780 | + | 3 |
| circFUNDC1 | chrX | 44383248 | 44386611 | - | 3 |
| circMIER1 | chr1 | 67423742 | 67428843 | + | 3 |
| circSSR1 | chr6 | 7303783 | 7310262 | - | 2 |
| circWDR60 | chr7 | 158662546 | 158669382 | + | 3 |
| circRBM33 | chr7 | 155465561 | 155473602 | + | 11 |
| circMAN1A2-1 | chr1 | 117944808 | 117957453 | + | 2 |
| circMAN1A2-2 | chr1 | 117944808 | 117963271 | + | 9 |
| circNAP1L4 | chr11 | 2972489 | 3000467 | - | 3 |
| circBPTF | chr17 | 65941525 | 65972074 | + | 2 |
| circMAN1A2-3 | chr1 | 117944808 | 117984947 | + | 2 |
| circCLTC | chr17 | 57721637 | 57763169 | + | 0 |
| circCDK11B | chr1 | 1586823 | 1650894 | - | 40 |

**Sup. Table 4** The significant GO term for the parental genes of each RBP’s bound circRNAs

| RBP | Tissue/cell line | GO term | adjusted-p | # parental genes |
| --- | --- | --- | --- | --- |
| FMR1 | HEK293 | PID_MYC_ACTIV_PATHWAY | 9.64×10^-6^ | 131 |
| PTBP1 | Hela | REACTOME_METABOLISM_OF_CARBOHYDRATES | 8.72×10^-6^ | 150 |
| PTBP1 | Hela | REACTOME_REGULATION_OF_GLUCOKINASE_BY_GLUCOKINASE_REGULATORY_PROTEIN | 3.04×10^-5^ | 150 |
| TRA2B | MDA-MB-231 | PID_AURORA_B_PATHWAY | 7.45×10^-7^ | 124 |
| DDX21 | HEK293 | REACTOME_METABOLISM_OF_PROTEINS | 3.36×10^-5^ | 105 |
| DDX21 | HEK293 | REACTOME_INFLUENZA_LIFE_CYCLE | 3.41×10^-6^ | 105 |
| YBX1 | MDA | KEGG_FOCAL_ADHESION | 1.29×10^-7^ | 551 |
| YBX1 | MDA | PID_FAK_PATHWAY | 1.98×10^-5^ | 551 |
